# Urethrotomia Externa

**Published:** 1881-09

**Authors:** Herman Mynter


### Clinical Reports.

### Urethrotomia Externa.

By Herman Mynter, M. D.

Mr. P. T. consulted me in August, 1881, for difficulties in passing his water. He is 52 years of age, a strong man, who, with the exception of the local difficulty, always has enjoyed good health. In his younger days he had two attacks of gonorrhœa. For 15 years he has passed his urine in a very small stream, and twice, respectively 8 and 6 years ago, had perfect retention of urine, which was relieved by hot baths. Instruments of moderate size have been introduced occasionally, but with difficulty, and the introduction was always followed by attacks of chills, fever and great prostration. He has twice (1 and 3 years ago) had abscesses form in the perineum, and the last abscess resulted in the formation of a urinary fistula. For months he has been unable to do any work, the water passing almost continually, both through the urethra and the fistula, drop by drop; his health had failed, he complains of pain over the kidneys and increasing weakness.

At the examination a hard callous fistula was seen behind the testicles in the scrotum, which could be followed about one inch upward toward the membranous part of the urethra. By examination through the urethra a stricture was found in the anterior part of the membranous urethra, hard and cicatrized, but no instrument could be passed through. The examination was followed by a severe attack of urethral fever. The patient was advised to submit to operative treatment, urethrotomia interna, if possible, if not urethrotomia externa. For some days he took grain doses of sulphate quinine three times a day and drop doses of tincture aconite. The operation was performed August 11th, Drs. Lothrop, Diehl, Storck, Cary and Davidson being present and assisting. The patient was put under the influence of ether, placed in the position for lithotomy, and hands and ankles securely fastened together. With a large metal syringe the urethra was injected with sweet oil with considerable power, and different-formed small bougies, belonging to Meissonenoe's instrument for internal urethrotomia, introduced, but in spite of all trials no one would pass the stricture. At last I succeeded in passing a very fine whale-bone bougie. A grooved sound for lithotomy was introduced down to the stricture, and held immovably against it. The end of it could be distinctly felt in the perineum. The scrotum was drawn well forward, the perineum divided in the middle line, and the urethra opened on to the sound in part of the stricture.

Two sutures were inserted in the walls of the urethra, and with sharp hooks the opening in the urethra made as wide as possible; but in the deep wound it was impossible to see anything, although the whale-bone bougie could be felt with a sound. The middle part of the bougie was therefore hooked with a strabismus hook and brought out through the wound, and with this as a guide, it was extremely easy to pass a brass grooved sound through the stricture and freely rinse the stricture. A large steel sound (No. 23, French scale) could then with ease be introduced into the bladder. The patient was put to bed, quinine in 10-grain doses and tincture aconite in 5-drop doses administered four times a day, and an injection of morphine (grain ¼) given subcutaneously. The wound was covered with towels dipped in warm carbolic acid lotion. The patient did not get any chill after the operation, and felt perfectly well. He was kept quiet in bed, the large steel sound was introduced once with ease every third day. The water passed partly through the wound for six days, but after that time only through the urethra, and the wound is now, fourteen days after the operation, almost healed, and the patient dismissed with the injunction to introduce the sound twice a week for years to come.

