# Errata in the September Number

**Published:** 1881-09

**Authors:** 


### ERRATA IN THE SEPTEMBER NUMBER.

Page 68, sixteenth line from below, for "grain doses" read "five-grain doses."
Eighth line from below for "Meissonenoe's" read "Meissoneuve's."
First line from below, "on to the sound in part of the stricture," read "upon the sound in front of the stricture."

Page 69, eighth line from above, "brass grooved" read "fine grooved."
Eighth line from above, "rinse" read "incise."

